# Mining Candidate Genes and Identifying Risk Factors for Leg Disease in Broilers: A Mendelian Randomization Study

**DOI:** 10.3390/ijms25168890

**Published:** 2024-08-15

**Authors:** Xinxin Tang, Peihao Liu, Na Luo, Jie Wen, Hegang Li, Guiping Zhao, Bingxing An

**Affiliations:** 1 Institute of Animal Sciences, Chinese Academy of Agricultural Sciences, Beijing 100080, China; tangeliauk@163.com (X.T.); lph112124@163.com (P.L.); itslorna@163.com (N.L.); wenjie@caas.cn (J.W.); 2 College of Animal Science and Technology, Qingdao Agricultural University, Qingdao 266109, China; 201701018@qau.edu.cn; 3 Center for Quantitative Genetics and Genomics (QGG), Aarhus University, 8000 Aarhus, Denmark

**Keywords:** white-feathered broilers, leg disease, bone traits, serum indicators, Mendelian randomization (MR)

## Abstract

Clinical investigations have highlighted disruptions in bone metabolic processes and abnormal fluctuations in serum indicator levels during the onset of leg disease (LD) in broilers. However, the presence of a genetic causal relationship for this association remains undetermined. Therefore, the aim of this study is to discern the risk factors underlying LD development using 1235 sequenced white-feathered broilers. We employed Mendelian randomization (MR) analysis to assess the associations of bone strength (BS), bone mineral density (BMD), tibial bone weight (TBW), tibial bone length (TBL), tibial bone diameter (TBD), bone ash (BA), ash calcium (Ash Ca), ash phosphorus (Ash P), serum calcium (Ca), serum phosphorus (P), serum alkaline phosphatase (ALP), and serum osteoprotegerin (OPG) with the incidence of LD. Compelling evidence underscores a causal link between the risk of developing LD and decreased BMD (odds ratio (OR) = 0.998; 95% CI: 0.983, 0.993; *P* < 0.001) and narrower TBD (OR = 0.985, 95% CI: 0.975, 0.994, *P* = 0.002). Additionally, serum OPG concentrations (OR: 0.995, 95% CI: 0.992, 0.999, *P* = 0.008) were associated with BMD (OR = 0.0078, 95% CI = 0.0043 to 0.0140, *P* < 0.001), indicating a robust genetic relationship between ALP concentrations (OR: 0.988, 95% CI: 0.984, 0.993, *P* < 0.001) and TBD (OR = 0.0046, 95% CI = 0.0026, 0.0083, *P* < 0.001). Moreover, elevated serum Ca (OR: 0.564, 95% CI: 0.487, 0.655, *P* < 0.001) and P (OR: 0.614, 95% CI: 0.539, 0.699, *P* < 0.001) levels were associated with a narrower TBD. Elevated serum levels of Ca, P, ALP, and OPG contribute to disturbances in bone metabolism, while decreased BMD and narrower TBD are associated with a greater risk of developing LD in broilers. This discovery elucidates the metabolic risk factors for LD in broilers and could provide information on LDs, such as osteoporosis, in humans.

## 1. Introduction

In broilers, leg disease (LD) is a chronic metabolic disorder that manifests clinically as impaired mobility, varying from minor gait instability to severe lameness or immobility [1]. This condition results in substantial economic losses and poses significant animal welfare concerns [2]. Leg disorders frequently afflict rapidly-growing broilers with high body weights, where bone growth lags behind the pace of weight gain, thereby disrupting the metabolic equilibrium [3,4]. This phenomenon is influenced by a myriad of factors such as genetics, nutrition, husbandry practices, environmental conditions, and even the incubation process [5]. This is evidenced by alterations in bone morphology (weight, length, diameter) and compromised bone quality (bone mineral density (BMD), bone mineral content, bone strength (BS), and bone ash (BA)), culminating in fractures, osteoporosis, and various other bone and leg disorders [6,7,8]. Deviant fluctuations in these parameters could be predisposing factors for the onset of bone leg disorders and serve as predictive indicators for assessing leg health and fracture risk. Nevertheless, the majority of these risk factors have been discerned through endophenotypic association studies, leaving uncertainties regarding their genetic causality.

Advances in sequencing technologies, including resequencing, simplified genome sequencing, and gene chips, have demonstrated the potential for genetic interventions to improve bone quality. This is evident through techniques such as genome-wide association studies (GWAS) and genomic selection, which have led to breakthroughs in the study of bone diseases and other conditions [9,10,11]. Building upon this foundation, Mendelian randomization (MR) studies utilizing genetic variation as instrumental variables (IVs) have gained widespread acceptance for their value in elucidating causal relationships between risk factors, such as biomarker levels, and disease outcomes [12]. Previous MR investigations in humans revealed associations between bone disorders and factors such as adiposity [13], inflammatory markers [14], uric acid [15], and immune dysregulation [16]. However, to date, there has been no systematic MR analysis from a metabolic perspective to evaluate the causal relationships between leg disorders, bone deterioration, and serum indicators. To address this gap, in the present study, we leveraged gene resequencing data from 1235 individual broilers and employed MR methods to investigate the potential causal relationship between the risk of developing LD and bone traits, as well as serum indicators. The aim of this study is to identify key risk factors for LD in broilers from a genetic perspective and to provide new insights into the metabolic molecular mechanisms underlying the occurrence of LD in broilers.

## 2. Results

**Figure 1:**
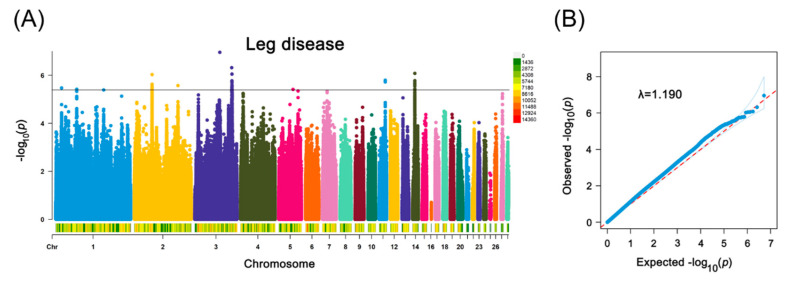
Manhattan (**A**) and Q–Q (**B**) plots of leg disease at the genome-wide level.

In the GWAS analysis, 5,259,518 LD-related SNPs were analyzed. Among these, 38 SNP loci distributed across chromosomes 1, 2, 3, 11, and 14 were identified as potentially significant at the genome-wide level (Figure 1A,B). Detailed information on the significant LD-related SNPs is provided in Table S1. The proportion of variance explained (PVE) by these loci ranged from 1.70% to 2.26%. Notably, the SNP locus rs65575230 on chromosome 3 reached a significance threshold of *P* < 1.98 × 10^−7^ (*P* = 1.12 × 10^−7^). This locus was annotated as a long intergenic noncoding RNA (lncRNA) and exhibited the highest PVE value (2.26%).

These significant SNP sites were annotated to five candidate genes: *MYBPC1*, *CDH13*, *CHTF18*, *MSLN*, and *ELMO1*. KEGG analysis did not reveal enrichment of important metabolic pathways. In the GO enrichment analysis, the top 20 significantly enriched GO terms, as depicted in Figure S1, included 40 terms that reached statistical significance (*P* < 0.05). These terms mainly encompass processes such as cell differentiation, biosynthesis, and regulation of signaling pathways. Among them, Rac protein signal transduction (GO:0016601) was the most significant (*P* = 4.22 × 10^−6^), with the *CDH13* and *ELMO1* genes showing notably high enrichment for this functional annotation.

The descriptive statistics of the bone and serum traits are presented in Table 1. Compared with healthy broilers, broilers with LD exhibited markedly lower values for bone parameters including BS, TBW, TBL, TBD, BA, Ash Ca, and Ash P (*P* < 0.05). Additionally, the serum levels of Ca and ALP were significantly lower in broilers within the LD group than in healthy broilers (*P* < 0.05), while there was an increasing trend in the levels of P and OPG.

Bone traits including bone BS, TBW, TBL, TBD, BA, Ash Ca, and Ash P were subjected to GWAS analysis, and the resulting Manhattan plots are shown in Figure S2. The computed inflation factors (λ) for each phenotype ranged from 0.939 to 1.092, indicating the accuracy and reliability of the GWAS results. A total of 214 broilers were included in the GWAS analysis for serum differential indicators including Ca, P, ALP, and OPG, and the resulting Manhattan plots are depicted in Figure S3. IVs were screened with the criterion of *P* < 10^−5^, resulting in 744 SNPs for bone traits and 114 SNPs for serum indicators. The F-statistics of these SNPs were all greater than 10, indicating a relatively low probability of weak instrumental bias, thus suggesting that the IVs were sufficiently plausible [17]. These compliant IVs were then overlaid with LD GWAS data. Following the exclusion of multiple SNPs associated with confounding factors, a total of 89 bone-related IVs were retained, comprising 3 SNP_BS_, 47 SNP_BMD_, 4 SNP_TBW_, 5 SNP_TBL_, 19 SNP_TBD_, 8 SNP_Ash Ca_, and 3 SNP_Ash P_. Additionally, 52 serum-related IVs, including 13 SNP_Ca_, 15 SNP_P_, 19 SNP_ALP_, and 5 SNP_OPG_, were eligible for inclusion in the MR analysis as exposure variables (Table S2). The F-statistics of these SNPs exceeded 10, indicating a reduced likelihood of weak instrumental bias and thus justifying the plausibility of the IVs.

**Figure 2:**
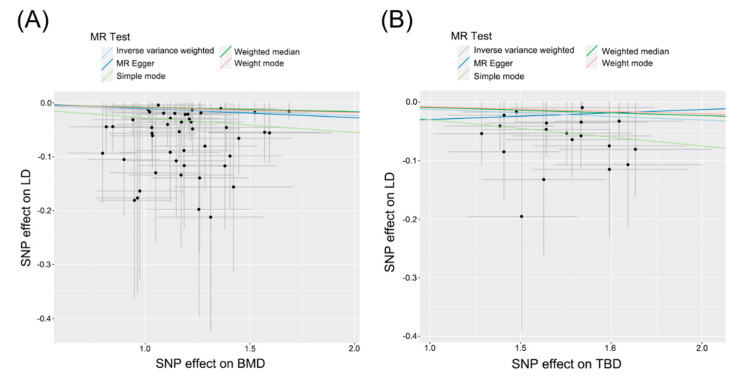
Scatter plots for MR analyses of the causal effect of (**A**) BMD and (**B**) TBD on LD. BMD: bone mineral density; TBD: tibial bone diameter; LD: leg disease.

The MR analysis revealed that diminished BMD (OR: 0.988, 95% CI: 0.983, 0.993, *P* < 0.001) and decreased TBD (OR: 0.985, 95% CI: 0.975, 0.994, *P* = 0.002) in bone traits were associated with an elevated risk of LD development in broilers (Figure 2A,B). The results of the heterogeneity and horizontal pleiotropy tests for the IVs for BMD and TB suggested the absence of heterogeneity or horizontal pleiotropy (*P* > 0.05). Sensitivity analyses utilizing the leave-one-out method demonstrated that the IVW estimates were not influenced by individual SNPs, confirming the reliability and validity of those results. Modeling outcomes further indicated no direct causal relationship between genetically predicted levels of Ca, P, ALP, OPG, and LD (Table S3).

Based on the above MR findings, a bidirectional MR analysis was employed to investigate the causal relationships between BMD, TBD, and serum Ca, P, ALP, and OPG. When BMD and TBD served as outcome variables, the IVs related to the serum indicators were juxtaposed with their respective GWAS data. Notably, in the GWAS data for serum P, the rs314201062 and rs316831956 loci on chromosome 2 and the rs317919754 locus on chromosome 17 were identified as being associated with confounding factors. Following the exclusion process, 22 SNP_Ca_, 27 SNP_P_, 44 SNP_ALP_, and 18 SNP_OPG_ were retained, resulting in 111 valid IVs for subsequent analyses. The F-statistics of these SNPs consistently surpassed 10, underscoring robust associations between these SNPs and the exposure variables, thus ensuring the reliability of SNP selection. Elevated OPG levels (OR: 0.995, 95% CI: 0.992, 0.999) were found to correlate with decreased BMD (*P* < 0.05), while greater serum Ca (OR: 0.564, 95% CI: 0.487, 0.655), P (OR: 0.614, 95% CI: 0.539, 0.699), and ALP (OR: 0.988, 95% CI: 0.984, 0.993) levels were associated with a reduction in TBD (*P* < 0.05).

**Figure 3:**
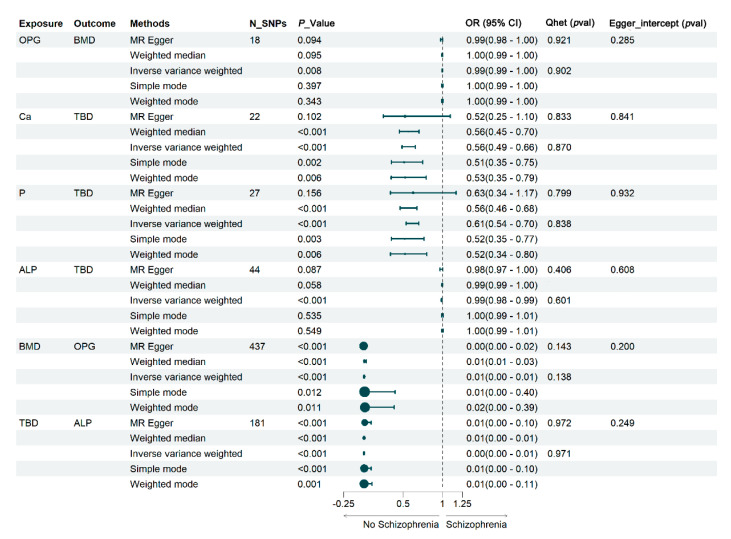
Forest plot showing estimates of causal effects between different bone traits and serum indicators. Each effect represents the estimated causal change in BMD, TBD, OPG, and ALP per SD change in OPG, Ca, P, ALP, BMD, and TBD. N_SNPs: number of SNPs used as genetic instruments in MR analyses; OR: odds ratio; CI: confidence interval.

When Ca, P, ALP, and OPG were considered as outcome variables, and BMD and TBD as exposure factors, the same methodology was applied, incorporating 437 SNP_BMD_ and 181 SNP_TBD_, resulting in a total of 618 SNPs used as IVs for MR analysis. MR analysis revealed significant associations between serum levels and the risk of bone damage. According to the IVW results, decreased BMD (OR = 0.0078, 95% CI = 0.0043–0.0140, *P* < 0.001) emerged as a risk factor for elevated serum OPG levels, while TBD exhibited a strong negative association with serum ALP (OR = 0.0046, 95% CI = 0.0026–0.0083, *P* < 0.001). Notably, the Cochran Q test using IVW detected no evidence of heterogeneity, and the MR–Egger intercept test indicated no presence of pleiotropy (Figure 3). Moreover, the sensitivity analysis employing the “leave-one-out” approach demonstrated that the causal associations between bone traits and serum indicators were not affected by any single SNP, confirming the validity and reliability of the analysis.

## 3. Discussion

The predominant cause of LD in contemporary broilers stems from compromised bone quality and incomplete calcification due to disruptions in bone metabolism, which in turn leads to fractures, osteoporosis, osteoarthritis, and other bone ailments [18], accompanied by aberrant changes in various serum biochemical factors [6,19]. Similar findings were reported in this investigation; however, the precise etiology and pathogenesis of broiler LD remain elusive. Furthermore, the understanding of its molecular regulatory mechanisms remains limited, with no substantiated evidence supporting the genetic influence on bone and serum traits in the development of broiler LD. In this study, we performed deep sequencing of the genomic regions of the broiler population and detected variants using resequencing technology. By identifying genetic variants associated with leg disease, we revealed their relationship with the phenotypic characteristics of the disease. This approach aims to provide preventive measures, diagnostic tools, and treatment options for the disease. Based on the resequencing data, we designed a case-control trial to elucidate the causal relationship between skeletal and serum indexes and leg disease (LD) in broiler chickens from a genetic perspective. This was achieved using small-scale GWAS data and magnetic resonance analysis. Therefore, this study was designed as a case-control trial leveraging small-scale GWAS data to elucidate the causal relationship between bone and serum indicators and broiler LD through the lens of genetics, employing MR analysis.

The GWAS results on LD pinpointed 38 SNP loci associated with the condition, including nine loci on chromosome 2, such as rs431841338 and rs431902025, which are linked to the gene *ELMO1*. This suggests the presence of multiple variant forms of this gene. *ELMO1* is a cytoplasmic bridging protein, and previous research has indicated that its deletion reduces osteoporosis in instances of OPG deficiency, ovariectomy, and inflammatory arthritis in mouse models. Additionally, *ELMO1* has been recognized as a signaling hub regulating osteoclasts and bone loss [20]. The gene *CDH13* encodes a calcineurin that diminishes osteoclast differentiation by inhibiting the NF-κB ligand receptor activator of kinase (RANKL) signaling pathway in bone marrow-derived macrophages. This disruption of bone remodeling homeostasis promotes bone loss [21]. These findings suggest their potential relevance to the biological processes and physiological functions underlying bone disease, rendering them plausible candidate genes for broiler LD. However, there is a dearth of studies linking the genes *MYBPC1*, *CHTF18*, and *MSLN* to bone growth and metabolism processes or calcium and phosphorus regulation. These associations warrant further exploration through experimental studies.

In this study, we observed negative associations of BMD and TBD with the risk of developing LD in broilers. These negative associations are likely closely linked to the mechanism of LD. For instance, damage to the bone structure may result in uneven force distribution on the legs, with reduced bone density and narrowed bone width increasing the risk of developing LD. The strong correlations between tibia length and width and metabolic characteristics such as organ size and bone muscle mass can be used as indicators of the degree of active bone metabolism during the growth and development of the organism [22,23]. BMD serves as a clinical assessment criterion for osteoporosis risk and bone fractures. Some researchers have employed large-scale data and MR methods to explore the causal relationship between BMD and osteoarthritis (OA), finding a significant association between OA and genetically predicted reductions in BMD. This suggests that measures to enhance BMD may effectively prevent OA [24]. Early animal experimental models have shown that decreased BMD leads to increased bone remodeling but insufficient mineralization, exacerbating cartilage damage, increasing the rate of bone loss, and ultimately contributing to the occurrence of OA [25,26,27]. A meta-analysis of 19 medications used in osteoporosis treatment yielded similar conclusions: a 2% or 6% increase in total BMD corresponded to a 28% or 66% reduction in fracture risk, respectively [28]. In light of the aforementioned studies, we posit that broiler LD arises as a consequence, rather than a precursor, of diminished BMD and narrowed TBD.

From the two-stage MR analysis, it is evident that serum OPG levels are causally related to BMD, while Ca, P, and ALP levels are causally linked to TBD. Serum factors play a regulatory role in LD by influencing bone metabolic processes and serving as predictive indicators for monitoring leg health. There is mounting evidence that specific serum biochemical factors, including parathyroid hormone (PTH), osteocalcin (OCN), and triglycerides (TG), are closely linked to disorders arising from bone development and metabolism [29,30]. In the context of type 2 diabetes mellitus (T2DM), diabetic bone disease is regarded as a late complication with a causal impact on increased BMD. Elevated serum levels of OPG effectively indicate that this process is marked by reduced bone turnover and diminished bone quality [8,31,32]. Inadequate intake of Ca, P, vitamins, and other nutrients hampers bone growth, development, and mineralization processes, culminating in decreased BMD and greater fracture susceptibility [33]. Extensive cohort and cross-sectional studies have established a causal link between elevated serum ALP levels and diminished BMD, impairing bone growth and development. Furthermore, higher ALP levels are significantly correlated with increased mortality risk due to various complications, elevated fracture incidence, cardiovascular disease, and other adverse events [34,35]. All of the aforementioned studies robustly underpin the findings of this examination. However, there is currently no evidence supporting a causal relationship between TBW, TBL, BS, BA, Ash Ca, or Ash P, and serum Ca, P, ALP, or OPG and LD. This suggests that these factors may not be the primary contributors to the pathogenesis of LD.

Interactions between bone metabolism and serum markers represent intricate biological processes. Bidirectional MR analysis revealed a reciprocal causal relationship between ALP and TBD, suggesting that ALP levels are not solely influenced by TBD, but may also impact the risk or progression of TBD. Moreover, OPG levels exhibited a negative bidirectional association with BMD, indicating that elevated OPG levels could lead to decreased BMD, while decreased BMD may, in turn, enhance OPG expression or activity. A study involving 6334 adults revealed a negative correlation between serum ALP and BMD, indicating that greater ALP activity is linked to increased bone turnover [36,37]. Another study revealed bone fragility and impaired osteoclastogenesis in a rat model with elevated ALP levels induced by bile duct ligation, leading to reduced BS [38]. This reciprocal inhibitory mechanism between BMD modifiers and BMD levels hints at complex biological mechanisms and interactions among ALP, OPG, TBD, and BMD. These bidirectional causal relationships may serve as potential biomarkers for bone LD by modulating ALP or OPG levels. Further investigation is warranted to elucidate the disease pathogenesis and aid in the early identification of high-risk individuals.

In this study, we systematically examined the associations between various bone and serum parameters and the risk of developing LD, focusing on bone metabolism. Elevated serum levels of Ca, P, ALP, and OPG were associated with decreased bone density and reduced width, contributing to an increased risk of developing LD in broilers. This discovery sheds new light on the potential mechanisms by which bone metabolism influences the development of LD in broilers. However, several limitations should be acknowledged. First, in this study, we evaluated only four relevant serum indicators, overlooking numerous other biochemical factors crucial for bone metabolism, such as CTx and FGF-23 [39,40]. Second, research on LD in broilers remains relatively scarce, with sample sizes considerably smaller than those of large-scale human disease cohorts. This scarcity may impede efforts to identify the genetic underpinnings of the disease, explore potential therapeutic avenues, and devise targeted preventive strategies. Third, current research on LD predominantly focuses on white-feathered broilers, which are characterized by higher growth rates and a greater incidence of leg ailments. However, it remains uncertain whether findings from such research are equally applicable to improving leg health in humans and other animals. Therefore, there is an urgent need for future research to address all of these limitations and to find solutions that will provide more effective ways to ameliorate leg diseases in humans and animals.

Since broiler leg diseases are closely related to metabolic processes, we can conduct a metabolomics analysis based on the data from this study. This analysis will compare molecular changes in metabolism between healthy and diseased broilers to understand the genetic basis of metabolites associated with leg diseases. By integrating metabolomics with genomic data, we aim to identify key genetic loci linked to signature metabolites and gain new insights into the genetic underpinnings of metabolic traits associated with broiler leg diseases. Our goal is to uncover the causes of leg disease and provide references for the prevention and targeted treatment of similar conditions in other species. This research aims to use metabolites as a focal point for understanding the causes of leg disease in broilers and to develop methods for prevention, early diagnosis, and targeted treatment, potentially benefiting other species as well.

## 4. Materials and Methods

### 4.1. Experimental Animals and Sample Collection

The present study utilized a cohort of 1235 “Guangming No. 2” white-feathered broilers with identical genetic backgrounds, comprising 1069 healthy broilers and 166 diseased broilers. All birds underwent uniform immunization protocols and were housed individually in cages measuring 30 × 25 × 45 cm (L × W × H), with ad libitum access to feed and water from 28 days of age. Blood, serum, and bone tissue samples were collected following a 12-h fasting period at 42 days of age to mitigate the influence of nocturnal fluctuations on measurements.

Sample collection: Two 2 mL blood samples were obtained by venous puncture of the wing tip. One whole blood sample from 99 healthy and 115 diseased broilers was left to stand for 5 h, centrifuged at 2000 r/min for 8 min, frozen in liquid nitrogen, and stored at −80 °C for subsequent serum biochemical analysis. The remaining tibia tissues were collected from 76 healthy and 99 diseased broilers, which were rinsed in 0.9% saline, wrapped in medical gauze, placed in self-sealing bags, and stored at −80 °C. All experiments were conducted in accordance with animal welfare standards. The experimental procedures involving animals were reviewed and approved by the Animal Experimentation Welfare Ethics Committee (IAS2022-37).

Phenotypic determination: Serum levels of Ca, P, ALP, and OPG were determined using the methylthymol blue (MTB) method, microplate method, phosphomolybdic acid (PAM) method, microenzymatic assay, and chicken OPG ELISA kit, respectively. Bone weight was measured using an electronic balance, while length and diameter were assessed with Vernier calipers. BS, BMD, and BA were determined using the three-point mechanical bending method, X dual-energy radiography, and the ash method (GB/T 6438-2007) [41], respectively. The Ca and P contents in the ash were analyzed using inductively coupled plasma emission spectrometry (ICP-ES). All measurements were conducted in strict accordance with the respective instrument protocols and kit instructions.

### 4.2. Data Resources

Firstly, we used the magnetic bead method to extract DNA from blood samples. Following fragmentation, end-repair, junction ligation, fragment amplification, cyclization, and DNB preparation, a library of 300–500 bp DNA fragments was constructed. Secondly, quality control and library testing ensured the suitability for loading onto the MGIDL-T 7 loading device, which then loaded the DNB onto the sequencing chip. Using the DNBSEQ-T 7 platform and joint probe anchored polymerization technology, 300–500 bp fragments were sequenced at an average depth of approximately 10 × 1 L. Upon sequencing, the raw data were filtered and the quality was assessed. Subsequently, the downstream raw data were subjected to read filtering using Fastp to eliminate low-quality reads, junctions, reads with more than 5 N bases, and reads shorter than 100 bases [42]. The remaining high-quality data were aligned to the chicken reference genome GRCg6a using the Sentieon tool embedded in the Burrows–Wheeler Aligner-MEM algorithm [43]. A total of 4378.31 Gb of sample sequencing output data (clean data) was generated, with an average of 11.93 Gb per sample and a mean Q30 value of 93.01% across all samples. Before genome-wide association analysis, loci with a minimum allele frequency ≥5% and genotyping rate ≥90% were retained from the genotypic data. Broilers with a genotyping rate <90% were excluded, and SNPs on sex chromosomes (Z and W chromosomes) were removed. After filtering, 5,259,518 autosomal variants were detected among the 1235 broilers. Among the 214 broilers with serum indicator phenotype data, there were 8,680,983 autosomal variants; while among the 175 broilers with bone trait data, there were 8,679,210 autosomal variants. Thirdly, the logistic regression model was employed for the GWAS analysis, treating LD as a binary variable distinguishing between health and disease. The parameter “-indep-Pairwise 25 5 0.2” in PLINK software was utilized to estimate the number of valid SNPs for independent tests. The resulting number of valid independent test SNPs was 252,645, thus establishing the potential significance threshold for GWAS analysis as 1/252,645 = 3.96 × 10^−6^ [44]. The contribution of annotated loci to phenotypic variation was assessed by calculating the phenotypic variation explained (PVE) [45]. Fourthly, the SNPs sourced from GWAS were annotated, followed by analysis of candidate genes using Gene Ontology (GO) and Kyoto Encyclopedia of Genes and Genomes (KEGG) functional enrichment analyses. This process aimed to elucidate the crucial biological functions of protein-coding genes. Fifthly, univariate linear mixed models, executed using GEMMA version 0.98.1 software, were constructed to analyze bone traits such as BS, BMD, TBW, TBL, TBD, BA, Ash Ca, Ash P, and the serum indicators Ca, P, ALP, and OPG via GWAS.

### 4.3. MR Analysis

MR analysis relies on utilizing genetic variants as IVs to infer causal relationships. In this study, SNPs associated with exposure were identified as IVs from GWAS results encompassing eight bone traits and four serum indicators. The use of valid IVs for MR analysis must simultaneously meet the following key assumptions.

Relevance assumption: Genetic variants must be correlated with the exposure variable of interest. In this study, SNPs associated with exposure were selected as IVs based on the criterion of *P* < 1 × 10^−5^. Independence assumption: Genetic variants should be independent of any confounding factors related to the outcome variable. SNPs in linkage disequilibrium with the aforementioned IVs were excluded using thresholds of r^2^ < 0.01 and kb < 10,000 to ensure IV independence [46]. Exclusion of limiting assumptions: Exclusivity, the effect of genetic variation on the outcome variable must be mediated solely through the exposure variable [47,48]. Allele-mismatched SNPs, palindromic SNPs, and missing SNPs were excluded from both the exposure and outcome variable effect comparisons. The validity of genetic variants as IVs was reasonably ensured based on the above three assumptions. Subsequently, the F statistic was employed to estimate the strength of the IVs’ effects, with only strong IVs (F > 10) retained for subsequent analysis.

The study was designed in two parts based on MR methodology. The first part employed unidirectional MR, using genetic variants associated with bone traits and serum indicators as IVs to infer direct causal relationships between those variables and LD incidence. In the second part, bidirectional MR was used to consider bone traits and serum indicators as reciprocal causal factors. Five methods, namely, MR–Egger, Weighted Median, Inverse Variance Weighted (IVW), Simple Mode, and Weighted Mode, were employed for MR analysis to better estimate the causal effects among the risk of developing LD, bone traits, and serum indicators levels. The IVW method is considered the standard MR analysis method, and it is based on the assumption that all IVs are valid; IVW involves combining the Wald ratio estimates of the IVs and selecting models for analysis based on the presence or absence of heterogeneity. Cochran’s Q test was used to determine the heterogeneity of the IVs on the outcome variables, with *P* > 0.05 indicating no heterogeneity among the SNPs. MR–Egger is used to test for potential pleiotropy by assessing whether the IVs affect the outcome through pathways other than exposure, with *P* > 0.05 for the regression intercept indicating the absence of horizontal pleiotropy. The Weighted Median requires that more than 50% of the IVs are valid SNPs. The Weighted Mode relies on small samples to ensure fewer biases and a lower type I error rate. The Simple Mode method can be used to group SNPs with similar estimated causal effects [49]. The impact of exposure on the outcomes was quantified using odds ratios (ORs) and their corresponding 95% confidence intervals.

To assess the reliability and validity of the MR results, the MR-pleiotropy residual sum and outlier (MR-PRESSO) method was used to detect and test outlier SNPs. Additionally, the “leave-one-out” method was employed to evaluate the influence of individual SNPs on the association between exposure and outcome variables. The MR analyses were conducted using the “Two Sample MR 0.5.7” package in R 4.2.3, with a significance level of α = 0.05. The results were visually presented using forest and funnel plots. The Mendelian randomization analysis flowchart is shown in Figure S4.

## 5. Conclusions

In this study, we designed a case-control trial to analyze the genetic basis of leg disease in broiler chickens, and annotated candidate genes *MYBPC1*, *CDH13*, *CHTF18*, *MSLN*, and *ELMO1* for the leg health phenotype. Compromised bone growth and disrupted metabolic processes are closely linked to the risk of developing LD. Through MR analysis, elevated serum levels of Ca, P, ALP, and OPG were found to decrease BMD and narrow TBD, consequently increasing the risk of developing LD in broilers. Moreover, a bidirectional causality is suggested between ALP and TBD, as well as OPG and BMD. The causal regulation of bone metabolism, serum biochemical indices, and leg health phenotypes was clarified, providing new insights to analyze the mechanism of leg disease in broiler chickens. Further investigations are warranted to assess the intricate relationship between bone metabolism and serum indicators, which could provide a foundational understanding for the comprehensive exploration of the genetic and metabolic mechanisms underlying LD.

## Figures and Tables

**Table 1:** Descriptive statistics of bone traits and serum indicators.

Traits	Total				Health	Leg Disease
	Mean ± Sd	CV (%)	Max	Min	Mean ± Sd	Mean ± Sd
BS (N)	334.43 ± 110.62	33	614.84	117.43	354.63 ± 104.46	319.89 ± 112.63
BMD (g/cm^3^)	0.15 ± 0.02	12	0.19	0.10	0.15 ± 0.014	0.15 ± 0.02
TBW (g)	15.77 ± 4.67	30	25.10	5.69	16.54 ± 4.59	15.19 ± 4.64
TBL (mm)	99.43 ± 10.77	11	116.20	71.04	101.56 ± 9.81	97.84 ± 11.17
TBD (mm)	7.21 ± 1.24	17	10.76	4.67	7.35 ± 1.38	7.10 ± 1.12
BA (g/g)	2.14 ± 0.53	25	3.80	0.90	2.30 ± 0.55	2.03 ± 0.48
Ash Ca (%)	34.20 ± 1.40	4	37.33	32.03	34.60 ± 1.53	33.90 ± 1.20
Ash P (%)	20.03 ± 0.61	3	21.24	19.05	20.23 ± 0.65	19.88 ± 0.53
Ca (mmol/L)	2.34 ± 0.46	19	3.54	1.43	2.28 ± 0.44	2.40 ± 0.46
P(mmol/L)	3.92 ± 0.52	13	5.14	2.30	3.98 ± 0.47	3.86 ± 0.56
ALP (Imperial unit/100 mL)	84.79 ± 42.11	50	234.62	26.35	87.54 ± 37.65	82.37 ± 45.54
OPG (pmol/L)	212.58 ± 51.80	24	375.90	98.26	222.30 ± 46.19	204.01 ± 54.87

BS: bone strength; BMD: bone mineral density; TBW: tibial bone weight; TBL: tibial bone length; TBD: tibial bone diameter; BA: bone ash; Ash Ca: ash calcium; Ash P: ash phosphorus; Ca: calcium; P: phosphorus; ALP: alkaline phosphatase; OPG: osteoprotegerin; Mean ± Sd: mean ± standard deviation; CV: coefficient of variation; Max: maximum value; Min: minimum value.

## Data Availability

All datasets generated for this study are included in the manuscript and its Supplementary Files.

## Supplementary Materials

The following supporting information can be downloaded at: https://www.mdpi.com/article/10.3390/ijms25168890/s1.
