# Supplementary material for: Mining Candidate Genes and Identifying Risk Factors for Leg Disease in Broilers: A Mendelian Randomization Study
Source: Int J Mol Sci. 2024 Aug 15;25(16):8890. doi: 10.3390/ijms25168890 (PMC11354539; doi:10.3390/ijms25168890)
Supplement: Supplementary file 1 [file ijms-25-08890-s001.zip › Supplementary figure.pdf]

FIGURE S1

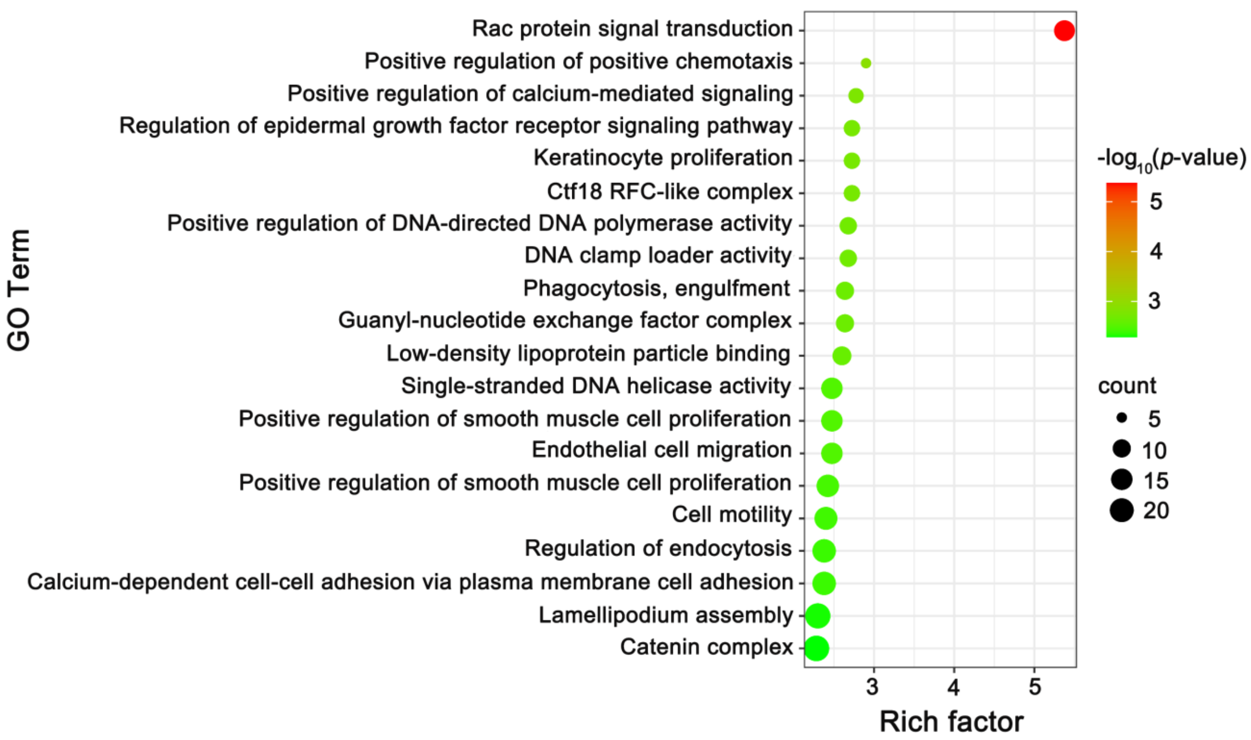

FIGURE S1 Top 20 significant pathways enriched in candidate genes for Gene Ontology (GO).

FIGURE S2

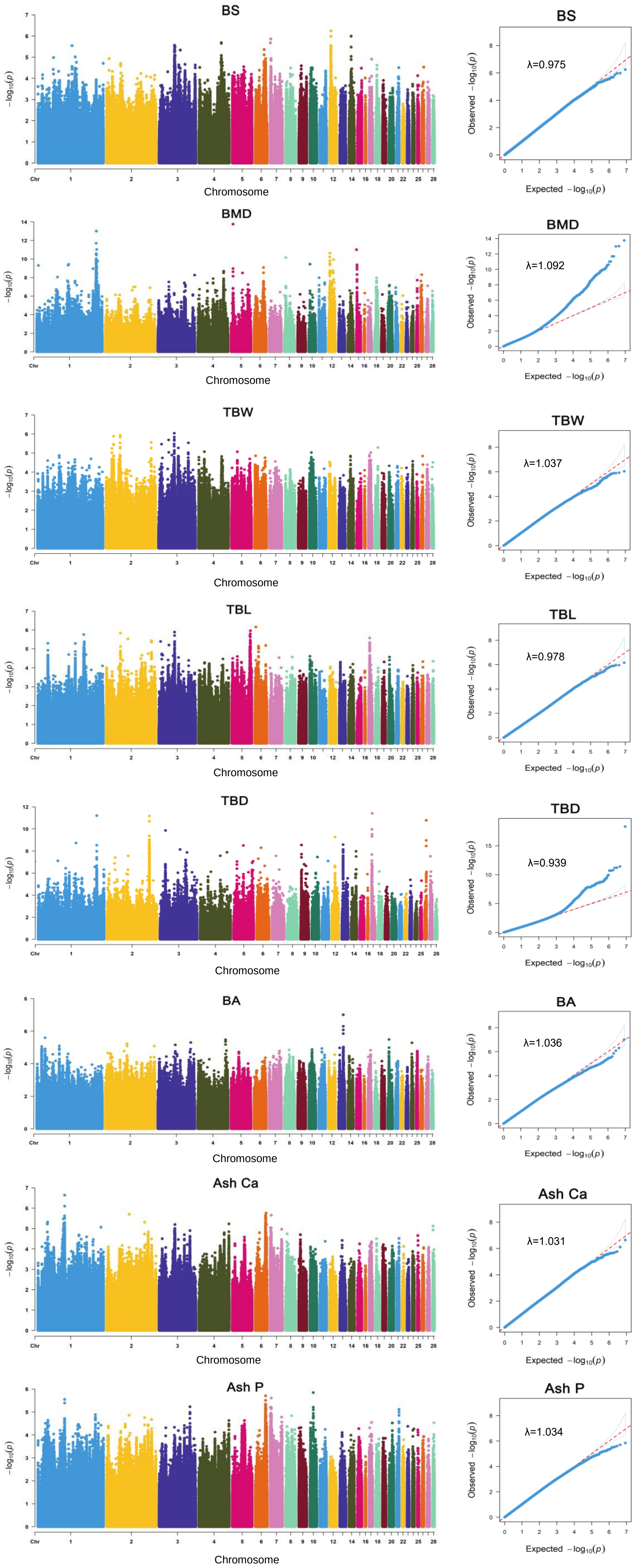

FIGURE S2 Manhattan plot and QQ-plot of BS, BMD, TBW, TBL, TBD, BA, Ash Ca, Ash P GWAS summary statistics

FIGURE S3

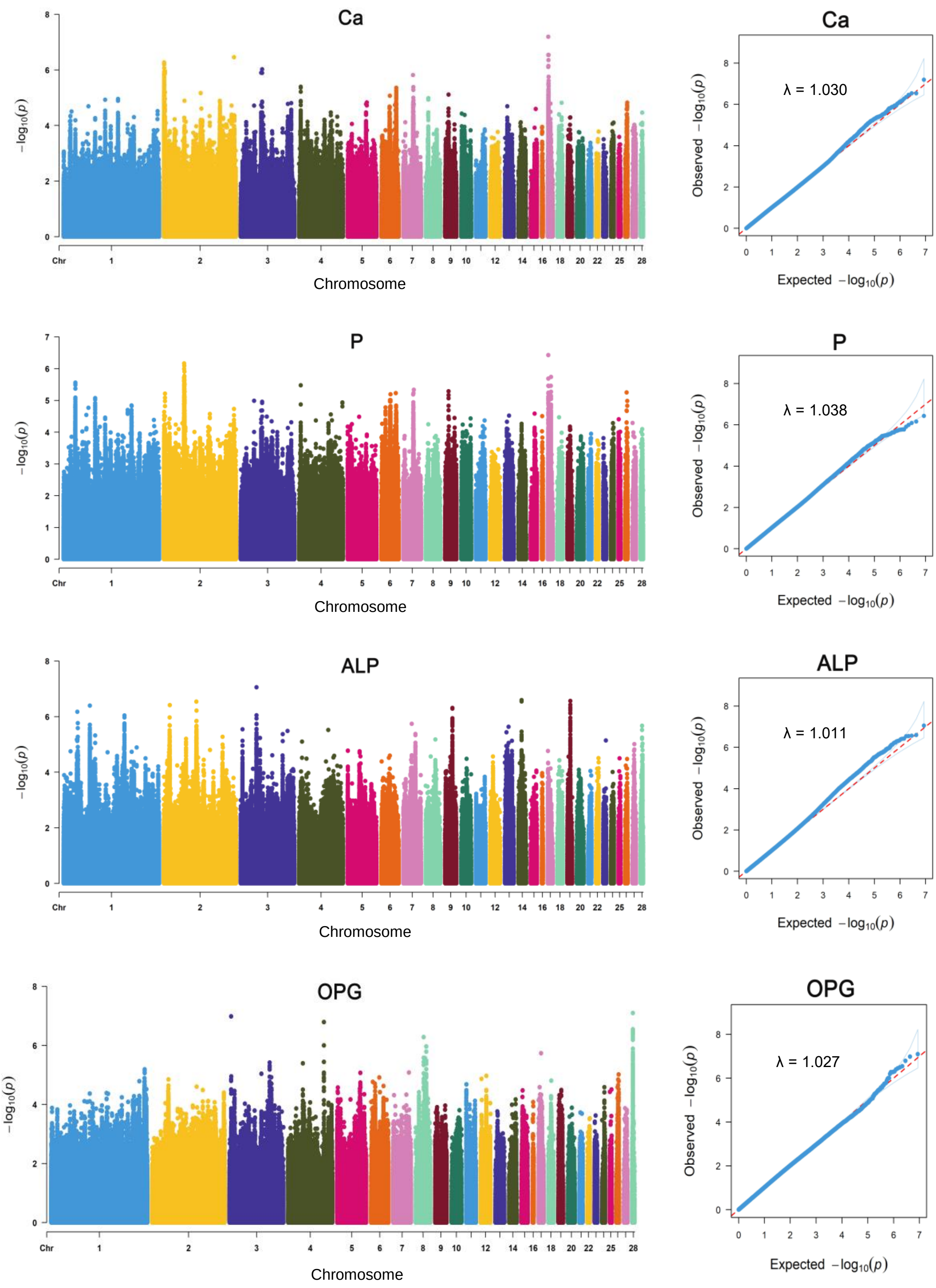

FIGURE S3 Manhattan plot and QQ-plot of Ca, P, ALP, OPG GWAS summary statistics

FIGURE S4

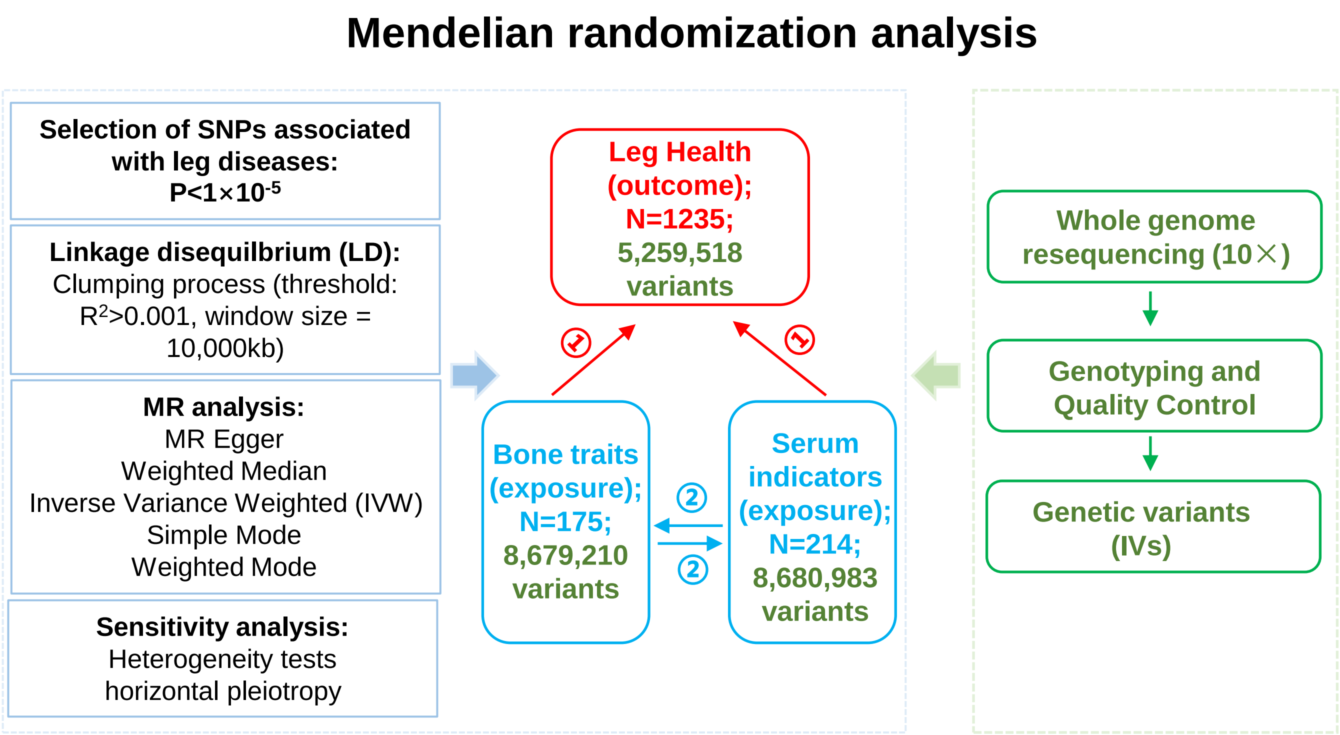

FIGURE S3 Schematic diagram of the Mendelian randomization process
